# Citrus Carotenoid Extracts Exert Anticancer Effects through Anti-Proliferation, Oxidative Stress, and Mitochondrial-Dependent Apoptosis in MCF-7 Cells

**DOI:** 10.3390/foods12183469

**Published:** 2023-09-18

**Authors:** Juanjuan Wei, Yurong Li, Zimao Ye, Yi Li, Zhiqin Zhou

**Affiliations:** 1 Key Laboratory of Agricultural Biosafety and Green Production of Upper Yangtze River (Ministry of Education), College of Horticulture and Landscape Architecture, Southwest University, Beibei District, Chongqing 400715, China; 18883375640@163.com (J.W.); yellor326815@163.com (Y.L.); zimaoye@163.com (Z.Y.); 2 Zhejiang Citrus Research Institute, Taizhou 318020, China; m_liyi@163.com; 3 The Southwest Institute of Fruits Nutrition, Banan District, Chongqing 400054, China

**Keywords:** carotenoids, citrus, reactive oxygen species (ROS), oxidative stress, apoptosis

## Abstract

Citrus is a globally popular fruit crop that contains bioactive compounds with numerous health benefits. Carotenoids are one of the main bioactive compounds present in citrus pulp. They possess exceptional antioxidant and anticancer properties, making them potentially effective in the prevention and treatment of breast cancer. Different citrus species, identified as ZMPG, DFGJ, NFMJ, XY, and ZHQC, were studied for their antioxidant activity and anticancer activity. XY had the highest total carotenoid content (75.30 µg/g FW), and ZHQC (ZH) had the lowest carotenoid content (19.74 µg/g FW). The composition of NFMJ, ZMPG, and DFHJ consisted of the most abundant number of carotenoids, while XY only had three types. The antioxidant capacity of the carotenoid extracts was evaluated, and ZH and DFHJ were identified as good sources of antioxidants. XY and ZH significantly inhibited cell proliferation, migration, and arresting cells during the G0/G1 phase. XY and ZH enhanced the accumulation of reactive oxygen species (ROS); reduced mitochondrial membrane potential (MMP); reduced the activities of antioxidant enzymes, including superoxide dismutase (SOD), catalase (CAT), glutathione reductase (GR), and peroxidase (POD); decreased glutathione (GSH) levels; and increased the malonaldehyde (MDA) content. Apoptosis occurred through the mitochondrial-mediated pathway through the up-regulation of BAX, caspase-3, and caspase-9 and the down-regulation of Bcl-2. In this study, the carotenoid-rich extracts of citrus pulp were found to induce oxidative stress through their pro-oxidant potential and regulate cell apoptosis in MCF-7 cancer cells. These results indicate that citrus carotenoids act as pro-oxidants and have the potential to be utilized for the development of anti-breast cancer products.

## 1. Introduction

Cancer, being the second leading cause of death globally, was responsible for about 9.6 million deaths in the year 2018. Breast cancer (BC) is the most common cancer in women worldwide. Due to its high morbidity and mortality, BC has become the top disease that threatens the lives of females, making it a global concern [1]. Because existing BC therapies are drug resistant, have side effects, and currently cannot completely cure BC, this poses a challenge to traditional therapeutics. Therefore, there is an urgent need to develop new treatments. Research studies have indicated that modifying one’s dietary habits through strategies such as increasing fruit intake can be an effective strategy in decreasing the occurrence of cancer or other severe illnesses [2]. Epidemiological studies have demonstrated a significant negative association between increased fruit intake and BC [3]. Plant foods have an inhibitory effect on the promotion and development stages of carcinogenesis, probably due to the significant concentration of bioactive phytochemicals [4]. In recent years, various phytocompounds derived from natural plants have been used for cancer therapy because of their significant pharmaceutical properties. Taxane, an FDA-approved chemotherapeutic drug, carries out a variety of anticancer functions, including disrupting the mitotic spindle and inhibiting tumor angiogenesis [5]. Resveratrol is a common polyphenol in plants and has been used as a pharmaceutical agent for the treatment of cancer, inhibiting cell invasion and metastasis in BC [6] and inhibiting lung cancer by targeting lung cancer stem-like cells [7].

Citrus fruits, which are favored by consumers for their flavors, colors, and nutritional qualities, constitute a significant portion of fruit cultivation, with an approximate worldwide output of 158.5 million MT [8]. Citrus fruits are widely studied by researchers because of their various biologically active functions, including their anticancer [9], antioxidant [10], anti-inflammatory [11], and anti-aging properties [12]. The primary reason for the biological effects observed in citrus fruits can be attributed to the presence of phytochemicals such as flavonoids, phenolic acid, dietary fiber, and carotenoids.

Carotenoids are a class of isoprenoid substances that are indispensable nutrients for humans due to their health-related benefits [13]. However, carotenoids must be uptaken through the diet (fruits and vegetables) since humans cannot synthesize them from scratch [14]. Citrus fruits contain the highest concentration of carotenoids among all fruits, making them a significant global source of dietary carotenoids [15]. There are about 20 kinds of carotenoids in tissues and blood, among which β-carotene, lutein, zeaxanthin, α-carotene, lycopene, and β-cryptoxanthin account for 60–70% of the plasma. Studies have shown that carotenoids contribute to vitamin A activity, as well as antioxidant, anti-inflammatory, and anticancer properties [14]. This is attributed to the mechanism related to the antioxidant and pro-oxidant behavior of carotenoids [16]. The antioxidant properties of carotenoids can protect normal cells from ROS-induced oxidative damage, thus serving as a preventive measure against cancer. The pro-oxidative activity of carotenoids induces oxidative damage to cancer cells, which can inhibit the proliferation of cancer cells. This is because oxidative stress restricts the progression and metastasis of cancer [16,17]. Pro-oxidants induce apoptosis by increasing the signaling pathway of ROS and/or weakening the antioxidant defense mechanism of malignant cells [18]. At present, pro-oxidants are favorable candidates the for selective targeting of tumor cells [19]. Therefore, plant-derived chemicals as pro-oxidants have broad prospects in cancer treatment, especially edible phytochemicals.

Studies have shown that carotenoid extracts from sweet potato peels have an inhibitory effect on breast cancer cells and tumors in mice [20]. Carotenoid-enriched extracts have inhibitory effects on the growth of human osteosarcoma cells and human colorectal adenocarcinoma cells [21]. Carotenoids, including β-carotene, lycopene, and lutein, have been extensively researched due to their notable anti-proliferation and pro-apoptotic effects (breast cancer, hepatic adenocarcinoma, and colon cancer) [22]. Animal model studies have demonstrated that the oxygenated carotenoid zeaxanthin can potentially lower the risk of specific types of cancers. Additionally, β-cryptoxanthin has been observed to effectively improve immune function and decrease inflammation [23,24]. Research has established a link between the activities of individual carotenoids and a decreased likelihood of developing cancer. Nevertheless, there remains a lack of detailed investigation into the potential health benefits of a diverse mixture of carotenoids from various citrus species. The mechanism of inhibiting/preventing the carcinogenic and mutagenic effects of carotenoids may be involved in radical oxygen species, the inhibition of cell proliferation, cell cycle arrest, and the regulation of signaling pathways, showing chemoprevention effects against BC. 

Considering the low toxicity of carotenoids and their ability to reduce the potential growth of cancer cells, more research is needed to further elucidate the effects of carotenoids on cancer occurrence and progression. Nevertheless, the lack of research on suitable cultivars and the correlation between phytochemicals and biological functions has restricted the development of citrus as a raw material for nutraceutical and food development. In this study, citrus pulp was chosen as the raw material for preparing the carotenoid extracts, and their composition and content were analyzed using HPLC. Three in vitro antioxidant capacity methods were used to evaluate the antioxidant activity of citrus carotenoids. At the same time, we examined the influence of citrus carotenoid extracts on cell proliferation, oxidation–reduction reactions, and ROS production, as well as their effects on cell cycle and apoptosis.

## 2. Materials and Methods

### 2.1. Plant Materials 

The five citrus materials we studied were the most commonly cultivated species from different places of origin. Four were from China, while the grapefruit used was from South Africa. Further details on the citrus materials used are shown in Table 1. For our analysis, the citrus fruits were divided into pulps and peels, which were immediately frozen in liquid nitrogen and ground into powder separately with a multifunctional pulverizer.

### 2.2. Reagents

Standards of β-carotene (98%) and lutein (99%) were purchased from Solarbio (Beijing, China). Standards of zeaxanthin (99%), lycopene (98%), β-cryptoxanthin (95%), and α-carotene (95%) were purchased from zzstandard (Shanghai, China). The samples used for our experiments were of HPLC grade; methanol, acetonitrile, and tetrahydrofuran (THF) were purchased from Sigma (St. Louis, MO, USA), while methyl tert-butyl ether (MTBE) was purchased from MREDA (Beijing, China). 2,4,6-Tris(2-pyridyl)-s-triazine (TPTZ), diammonium 2,2′-azino-bis (3-ethylbenzothiazoline-6-sulfonate) (ABTS), 2,2-diphenyl-1-picrylhydrazyl (DPPH), (±)-6-hydroxy-2,5,7,8-tetramethylchromane-2-carboxylic acid (Trolox), 3,4,5-trihydroxybenzoic acid, and rutin were purchased from Sigma (St. Louis, MO, USA). The other reagents used in the experiment were of analytical grade and obtained from Kelong (Chengdu, China).

DMSO, penicillin and streptomycin, enzyme activity assay kits (SOD, CAT and GR), content assay kits (MDA and GSH), and BCA protein assay kits were purchased from Solarbio Life Science and Technology Ltd. (Beijing, China). A JC-1 mitochondrial membrane potential fluorescent probe, purchased from Solarbio, was used at 10 µM in PBS. The cell cycle analysis kit (PI) and reactive oxygen species kit (DCFH-DA) we used were obtained from Biosharp (Hefei, China). The Annexin V-FITC/PI apoptosis kit was purchased from Elabscience (Wuhan, China). The Edu cell proliferation kit, giemsa stain, and 4% paraformaldehyde fix solution were obtained from Beyotime (Shanghai, China).

### 2.3. Extraction of Citrus Carotenoids

Citrus carotenoids were extracted according to a modified form of Kaijie Zhu and Rodriguez-Amaya’s method [25,26]. Briefly, the samples of citrus peel or pulp were extracted with 25 mL acetone/ethanol/hexane (1:1:2) and 0.1% BHT for 30 min under ultrasonic conditions. The process of carotenoid extraction was repeated three times, and the organic layer was merged. The organic layer was concentrated at 30 °C using a rotary vacuum evaporator. The concentrated extracts were dried in N_2_ and stored at −20 °C until use.

### 2.4. Identification of Carotenoids Using HPLC

The carotenoid extracts were saponified by adding 10% KOH–methanol solution (0.1% BHT) before detection. The mixture was removed from the dark, and 5 mL of water and 2 mL of MTBE were added to collect the organic layer; then, 4 mL of MTBE was added to wash the sample, and the organic layer was subsequently collected. The solution was further washed until void of alkali and subsequently concentrated and dried [27]. For the samples to be analyzed using HPLC, the solution needed to be filtered through a 0.22 µm filter. 

The carotenoid content was chromatographically quantified using HPLC-PDA (Waters, Milford, MA, USA). Carotenoids were determined using a C30 column (4.6 × 250 mm i.d., 5 μm, YMC, Kyoto, Japan). As previously described [28], the mobile phases were MTBE as eluent A, methanol as eluent B, and H_2_O as eluent C. A gradient program was used, where the initial condition was 60% B/40% C; 0–5 min, 80% B/20% C; 5–10 min, 15%A/81% B/4% C; 10–60 min, 85%A/11% B/4% C; 60–71 min, 100% B; 71–72 min, then back to the initial condition for re-equilibration. The flow was 1.0 mL/min, and the injection volume was 10 µL. The wavelength and oven temperature were set to 450 nm, 470 nm, and 27 °C, respectively.

The HPLC-DAD method combined with retention time (RT), UV-visible spectrum data, and co-injected standards was used for carotenoid identification. In addition, the spectral fine structure value III/II and the ratio of the absorbance of the peak in the UV-vis spectrum to the absorbance of the second absorption band in the visible region were compared with the literature to confirm the identification of carotenoid monomers [26,28]. Their relative contents were calculated based on the content of standard β-cryptoxanthin. 

### 2.5. Antioxidant Capacity

Antioxidant activity was measured using ABTS• + radical with minor modifications [10]. We adjusted the absorbance after diluting the stock solution to 0.70 ± 0.02 and taking 2.5 mL of the diluted solution and mixing it with 50 µL of the carotenoid extracts; subsequently, we allowed the mixture to react for 10 min in the dark. After the reaction, absorbance values were measured at 734 nm. The standard curve was linear between 0 and 1000 μM Trolox. The absorbance response (y) of Trolox was (y = −0.0005x + 0.6908, R^2^ = 0.9994), with liner concentrations. The antioxidant activity of the carotenoid extracts was calculated using the above formula and expressed as TEAC (µmol/g TE FW). 

The DPPH free radical scavenging activity was analyzed according to the method described in a previous study (with modification) [29]. In brief, 0.5 mL of the carotenoid extract was mixed with 3.8 mL of DPPH solution. After shaking, the reaction was left in the dark for 30 min, and the absorption was evaluated at a wavelength of 517 nm. The formula for determining the sample’s equivalent antioxidant activity was derived as y = −0.0025x + 0.6375 (R^2^ = 0.9990). The TEAC was used to represent the results.

Ferric reducing antioxidant power (FRAP) refers to the method reported by a previous study [30]. Briefly, the TPTZ solution comprised 20 mL of a 10 mM TPTZ solution in 40 mM HCl, 20 mL of 20 mM FeCl_3_, and 200 mL of 0.3 M acetate buffer (pH = 3.6). A total of 150 µL of carotenoid extract was added to 2.5 mL of the TPTZ solution. After mixing the liquid, the reaction was left for 30 min in the dark, and the absorbance was measured at 593 nm. The standard curve equation was y = 0.0024x − 0.00226, R^2^ = 0.9992, and the results were expressed as TEAC.

### 2.6. Cell Culture and Reagents

MCF-7 cells (human breast cancer cells) were obtained from Procell Life Science & Technology Co., Ltd. (Wuhan, China). The MCF-7 cells were cultured in Eagle’s medium (MEM) (Procell, Wuhan, China) containing 10% FBS (BI, Montevideo, Uruguay, South America), 1% penicillin-streptomycin, and 0.01 mg/mL insulin. The cells were cultured in a constant temperature incubator containing 5% CO_2_ at 37 °C. The cells were incubated for 24 h before treatment with the extracts.

### 2.7. Cell Survival Assay 

For in vitro evaluation, the MCF-7 cells were selected to test the different concentrations (0~200 µg/mL) of the extracts. The final concentration of DMSO in the culture medium was less than 0.5%. The cells were seeded in 96-well plates (1 × 10^4^ cells/well) under humidified conditions in 5% CO_2_ at 37 °C for 24 h. Then, the cytotoxicity of the different extracts was measured using a CCK8 assay. Experiments were repeated three times independently.

### 2.8. Edu Staining

Edu (5-ethynyl-2′-deoxyuridine), a novel thymidine analogue, can be incorporated into newly synthesized DNA instead of thymidine during DNA synthesis. After treating the MCF-7 cells with various concentrations of extract, they were thoroughly rinsed thrice using PBS. Next, the cells were exposed to a working solution of Edu (10 µM) and incubated in darkness for 4 h at a temperature of 37 °C. After incubation, the cells were washed with PBS three times and fixed with 4% paraformaldehyde for 15 min. Next, the cells were permeabilized with 0.3% TritonX-100 for 15 min and washed with PBS three times. The cells were then incubated with Hoechst 33342 for 10 min. The images were captured using a fluorescence microscope (Olympus U-HGLGPS, Shanghai, China).

### 2.9. Cell Cycle and Apoptosis Analysis

The MCF-7 cells (3 × 10^5^ cells/well) were seeded into 6-well plates and allowed to grow for 24 h. Following this, the cells were treated with DMSO or carotenoid extract for 48 h. After treatment, cell samples were obtained and washed with two rounds of cold 1 × PBS and then resuspended in 500 µL binding buffer. Next, the cells were labeled with Annexin V–FITC/PI to examine apoptosis. The treated cells were collected and washed twice with pre-cooled PBS and fixed with 70% ethanol. A 0.5 mL mix of propidium iodide (PI) with RNaseA was used to stain the cells at 37 °C for 30 min in the dark. The signals were collected using a flow cytometer (BD, PerkinElmer, Waltham, MA, USA), with 104 cells counted in each sample.

### 2.10. Wound Healing Assay

A cell scratch wound healing assay was mainly used to detect the invasion and migration ability of tumor cells. MCF-7 cells (1 × 10^6^ cells/well) were seeded in 6-well plates and cultured in a 5% CO_2_ incubator at 37 °C for 24 h. The cell monolayer was subjected to cross-scratches using 200 µL pipettes, followed by three rounds of PBS washes to eliminate any floating cells. The cells were then treated with various concentrations of carotenoid extract. At 0 h and 48 h, the treated cells were photographed using an inverted fluorescence microscope (Olympus U-HGLGPS, Suzhou, China). Their mobility was analyzed using 1.4.3.67 ImageJ software.

### 2.11. Analysis of the Mitochondrial Membrane Potential (MMP)

To detect the mitochondrial membrane potential of the MCF-7 cells, a mitochondrial membrane potential detection kit was used, following the instructions provided by the manufacturer. Cellular incubation was carried out at 37 °C in the absence of light for 20 min using the JC-1 working solution. Post incubation, the cells underwent two rounds of washing with the JC-1 buffer solution. Following this, fluorescence intensity measurements were acquired using a multimode microplate reader at an excitation wavelength of 490 nm and an emission wavelength of 530 nm. Additionally, the fluorescence microscope captured images of the cells after the aforementioned steps. JC-1 exhibits two emission wavelengths, red and green, and the reduction in the ratio of fluorescence intensity between the red and green emissions suggests an increase in mitochondrial membrane depolarization.

### 2.12. Measurement of ROS Level

The intracellular ROS levels in the MCF-7 cells were measured using 2′,7′-dichlorofluorescein diacetate (DCFH-DA). The DCFH-DA probe is taken up by cells, deacetylated to generate 2′, 7′-dichlorodihydrofluorescein (DCFH), and then rapidly oxidized to generate a strong fluorescent product 2′,7-dichlorofluorescein (DCF). Briefly, MCF-7 cells were cultured overnight in 96-well plates in 5% CO_2_ at 37 °C. After treatment, the cells were incubated with 10 µM DCFH-DA for 30 min at 37 °C. After incubation, the cells were rinsed with serum-free MEM. Fluorescent images of the cells were acquired using a fluorescence microscope (Olympus U-HGLGPS, Suzhou, China). 

### 2.13. Measurement of SOD, CAT, GR, and POD Activity, as Well as GSH and MDA Levels 

The levels of oxidative stress markers, including GSH and MDA, were detected following the instructions of the assay kits. The levels of antioxidant factors SOD, CAT, POD, and GR were separately determined using assay kits. The protein content was determined using the BCA method.

### 2.14. RNA Isolation and qRT-PCR

RNA was extracted from the cells according to the manufacturer’s protocol (TIANGEN, Beijing, China). Then, the isolated total RNA was reverse-transcribed into cDNA following the manufacturer’s instructions (Vazyme, Nanjing, China). Quantitative real-time PCR (qRT-PCR) analysis was performed using gene-specific primers on the Bio-Rad CFX 96 system. To quantify the PCR products, SYBR Green Master Mix (Vazyme, Nanjing, China) was utilized in accordance with the kit’s instructions. All reactions were normalized to GAPDH levels. The primer sequences are shown in Supplementary Table S1.

### 2.15. Statistical Analysis

The experiments were replicated threefold, and the results are presented as mean ± standard deviation (SD). Once homogenous differences had been confirmed, the data were tested using an ANOVA using GraphPad Prism 8.0.2 software, and significant differences between the groups were evaluated using Tukey’s test. Differences in the means were considered significant at *p* < 0.05 or *p* < 0.01.

## 3. Results

### 3.1. Characterization of the Main Carotenoid Compounds of the Cultivated Citrus 

**Figure 1:**
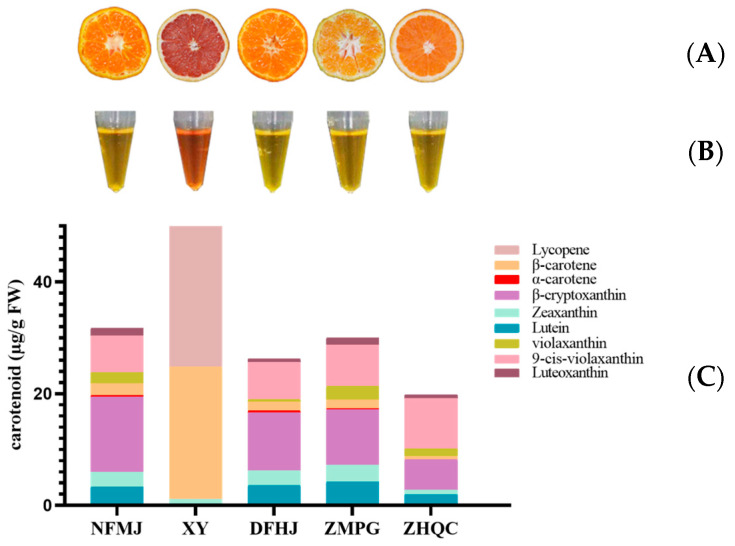
Content and composition of carotenoids in five citrus samples. (**A**) The phenotypes of five citrus fruits. (**B**) Colors of carotenoid extracts in five samples. (**C**) The difference in carotenoid content and composition of five citrus. DFHJ: carotenoid extract of DongFangHongJu; NFMJ: carotenoid extract of NanFengMiJu; ZHQC: carotenoid extract of ZaoHongQiCheng; ZMPG: carotenoid extract of ZaoMiPengGan; XY: carotenoid extract of XiYou.

The carotenoid content in the citrus fruits exhibits significant variability, and this has been well documented. After comparison with the RT, spectrum data, and characteristic peaks of the standards, a monomer was identified in the carotenoid extract, and this monomer is shown in Table 2. From Figure 1A, we can observe that citrus pulp exhibited different colors, which may be attributable to the different types and contents of carotenoids. Consistent with the color phenotype of the citrus pulp, the colors of the carotenoid extracts of these five citrus species were also different (Figure 1B). Further, the carotenoid composition and contents of five samples were analyzed (Figure 1C). In the carotenoids analyzed, the highest carotenoid content of XY was 75.30 µg/g, and the lowest content of ZHQC was 19.74 µg/g. In particular, the XY carotenoid extract mainly contained a large amount of β-carotene (23.87 µg/g) and lycopene (50.32 µg/g). Mandarin mainly contained β-cryptoxanthin, lutein, zeaxanthin, and 9-cis-violaxanthin, but the contents of α-carotene and β-carotene were very low. Interestingly, ZHQC had a similar composition to ZMPG, NFMJ, and DFHJ, but its carotenoid content was low. By conducting a comprehensive analysis, both qualitative and quantitative, on the carotenoids, we discovered a significant presence of carotenoids in the extracts of various citrus cultivars. However, variations were observed in terms of the quantity and composition of the primary constituents. 

### 3.2. Antioxidant Activity

The antioxidant capacity of various samples was assessed using the DPPH, ABTS, and FRAP methods, and the results are shown in Table 3. Among the different samples, the trends of the three methods for measuring antioxidant capacity were roughly the same. The range of DPPH radical scavenging in the samples was 1.07–2.44 µmol/g TE FW; the range of ABTS radical scavenging was 2.91–6.85 µmol/g TE FW, and the FRAP value range was 1.49–2.77 µmol/g TE FW. ZHQC showed the highest antioxidant activity, with a TEAC of 6.85 µmol/g, 2.44 µmol/g, and 2.77 µmol/g using the ABTS, DPPH, and FRAP, respectively. NFMJ had the lowest antioxidant capacity among the three different methods (1.07 µmol/g TE FW for DPPH, 2.91 µmol/g TE FW for ABTS, and 1.49 µmol/g TE FW for FRAP). In order to better evaluate the total antioxidant capacity of the samples, this study used the comprehensive antioxidant performance index (APC index) for evaluation, and the ranking of different samples is shown in Table 3. The APC index of the studied samples was 46.7–100%. According to the results of our investigation into the total antioxidant capacity of citrus carotenoid extracts, the ranking is as follows: ZHQC > DFHJ > ZMPG > XY > NFMJ. These results indicate that the carotenoid extracts of ZHQC and DFHJ are good sources of antioxidants.

### 3.3. Cytotoxicity of Carotenoid Extracts on Breast Cancer Cells

**Figure 2:**
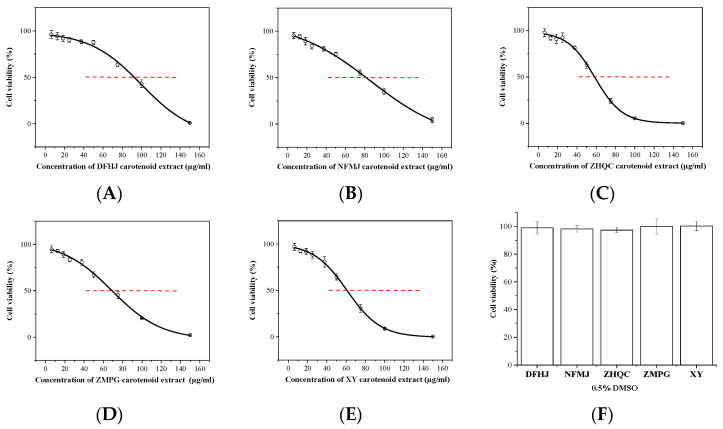
Effect of the cytotoxicity of different citrus carotenoid extracts on the growth of MCF-7 cells. (**A**–**E**) The viability of the MCF-7 cells was observed to decrease after 48 h of treatment with various concentrations of DFHJ, NFMJ, ZHQC, ZMPG, and XY carotenoid extract. (**F**) Treatment with 0.5% DMSO did not affect cell viability.

To explore the impact of carotenoid extracts on the growth of MCF-7 cells, we investigated cell viability after incubation with various concentrations (0, 6.25, 12.5, 25, 37.5, 50, 75, 100, and 150 µg/mL) for 48 h. The evaluation of cell viability was performed using the CCK8 kit. The control group was treated with 0.5% DMSO, and cell viability was not affected (Figure 2F). The viability of the MCF-7 cells was significantly decreased in a dose-dependent manner regardless of the carotenoid extract (Figure 2). The IC50 values of the five carotenoid extracts of DFHJ, NFMJ, ZHQC, ZMPG, and XY were 91.70 µg/mL, 86.17 µg/mL, 58.53 µg/mL, 68.78 µg/mL, and 61.34 µg/mL, respectively (Table 4). Specifically, the ZHQC and XY extracts showed a significant cytotoxic effect at a lower concentration. Therefore, the carotenoid extracts of ZHQC (ZH) and XY were used for our subsequent experiments at concentrations of 30 and 60 µg/mL.

### 3.4. Effects of Carotenoid Extracts on the Proliferation and Migration of MCF-7 Cells

**Figure 3:**
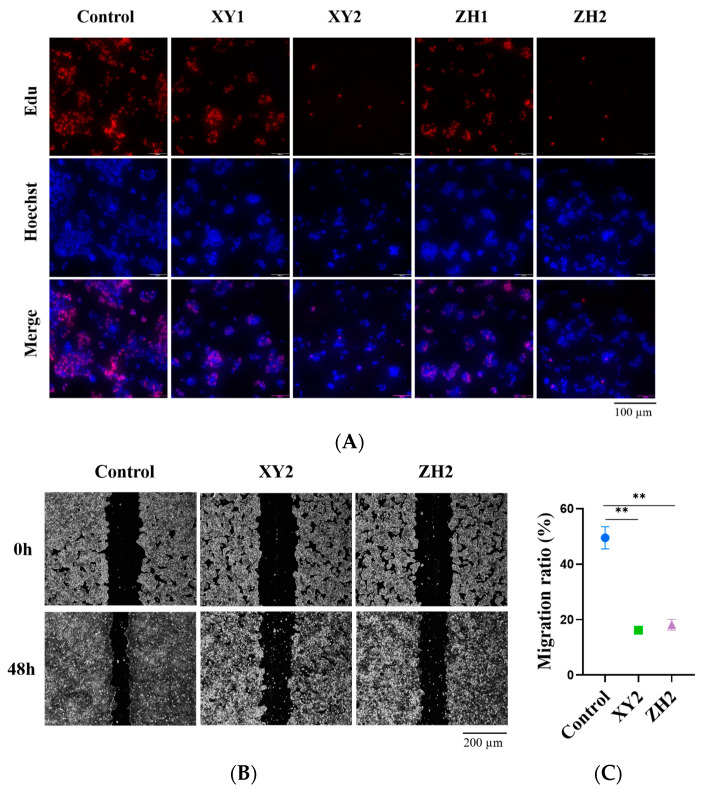
Carotenoid extracts of XY and ZH inhibit the proliferation and migration of breast tumor cells. (**A**) XY1, XY2, ZH1, and ZH2 inhibited MCF-7 cell proliferation. Cell proliferation and nuclei were stained using Edu kit and Hoechst 33342, respectively. (**B**) The wound-healing assay was employed to determine the cell migration ability of the MCF-7 cells that were subjected to serum-free medium conditions for 48 h and treated with concentrations of ZH2 and XY2. (**C**) Statistical data of migration ratio (%). Mean ± SD. ** *p* < 0.01. XY1 = 30 µg/mL, XY2 = 60 µg/mL, ZH1 = 30 µg/mL, ZH2 = 60 µg/mL.

To determine whether carotenoid extracts affected the cell proliferation of breast cancer cells, we further validated the suppressed effect of ZH and XY in cell proliferation using the Edu staining assay. The results of the Edu proliferation assay demonstrated that the red fluorescence, which represents the proliferation of MCF-7 cells, is inhibited by different XY and ZH treatments. Compared with XY1 and ZH1, XY2 and ZH2 significantly inhibited cell proliferation (Figure 3A).

The process of tumor metastasis is complex and consists of multiple steps. The early stage of the process includes the adhesion, migration, and invasion of tumor cells to the vascular endothelium, and the later stage involves the colonization of tumor cells in tissues or organs [31]. Thus, we studied the effects of ZH and XY on MCF-7 cell migration. We found that the cell rate of migration was markedly reduced after treatment with XY2 and ZH2 for 48 h (Figure 3B), and the migration ratio was 16.19% and 18.16% in the 60 µg/mL treatment group, respectively (Figure 3C). Compared to the control group, the migration ratio of the treatment group was reduced by more than 30%. Overall, our findings suggest that XY and ZH play important roles in inhibiting breast cancer cell proliferation and migration.

### 3.5. Effects of Carotenoid Extracts on the Cell Cycle

**Figure 4:**
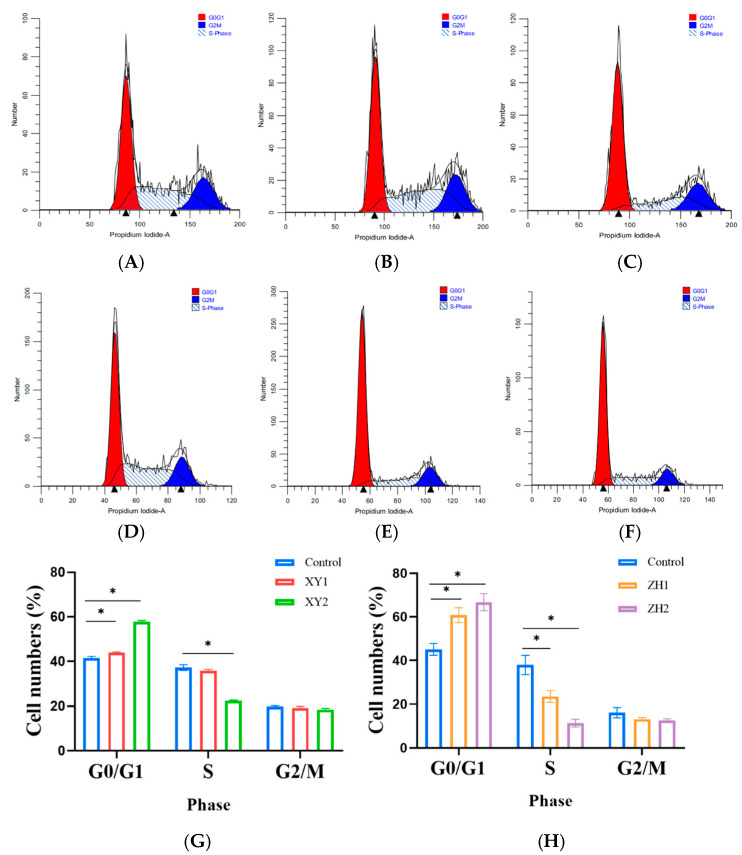
Effects of carotenoid extracts of XY and ZH on cell cycle phase distributions of MCF-7 cells. (**A**–**F**) Cell cycle distribution was determined via flow cytometry. (**A**) Control; (**B**) XY1; (**C**) XY2; (**D**) Control; (**E**) ZH1; (**F**) ZH2. (**G**,**H**) The proportion of cell population in G0/G1, S, and G2/M phase. Mean ± SD. * *p* < 0.05.

Interfering with the cycle of cancer cells and blocking the interphase of cancer cells is an important target for the activity of anticancer drugs [32]. The results of the cytotoxicity assay and Edu staining showed that XY2 and ZH2 could markedly inhibit the proliferation of MCF-7 cells. Therefore, we speculate that XY and ZH may affect cell proliferation by interfering with the cell cycle. As shown in Figure 4, PI staining was performed on the MCF-7 cells treated with XY1, XY2, ZH1, and ZH2 for 48 h; after staining, flow cytometry analysis was performed. The number of cells in the G0/G1 phase increased in a dose-dependent manner; however, the number of cells in the G2/M phase were reduced. The statistical data indicated a significant increase in the percentage of cells in the G0/G1 phase in the XY2 and ZH2 treatments (Figure 4G,H). The results show that XY and ZH induced G0/G1 arrest in the MCF-7 cells.

### 3.6. Effects of Carotenoid Extracts on Cell Apoptosis

**Figure 5:**
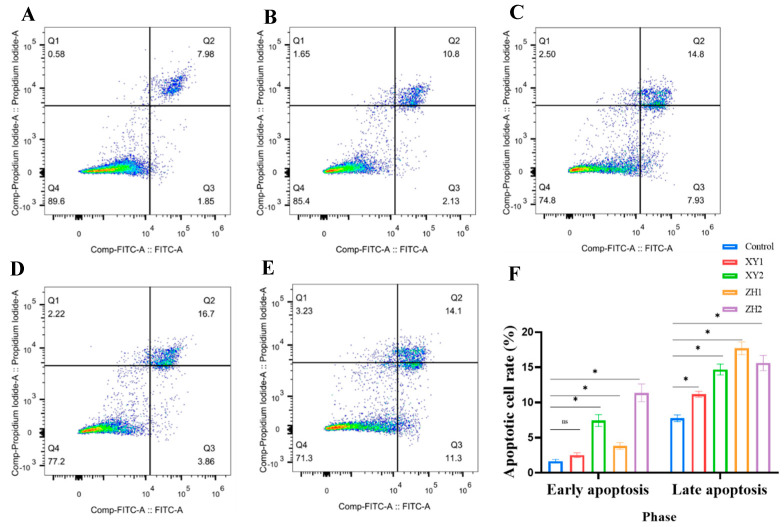
Effects of carotenoid extracts of XY and ZH on the cell apoptosis of MCF-7 cells. (**A**–**E**) The apoptotic cell is divided into four quadrants after Annexin V-FITC/PI staining; necrotic cells are assigned to Q1, late apoptotic cells to Q2, early apoptotic cells to Q3, and viable cells to Q4. (**A**) Control; (**B**) XY1; (**C**) XY2; (**D**) ZH1; (**E**) ZH2. (**F**) Data regarding early apoptosis rate and late apoptosis rate. Mean ± SD. * *p* < 0.05.

In order to investigate the effects of XY and ZH on the anticancer properties of the MCF-7 cells, we used flow cytometry to detect the results of MCF-7 cell apoptosis, aiming to reveal more details on the mechanisms. Figure 5 shows the percentages of living, early apoptotic, and late apoptotic MCF-7 cells after incubation with XY and ZH for 48 h. Compared with the control group, 60 μg/mL of XY2 and ZH2 significantly increased the apoptosis of MCF-7 cells by 22.73% and 25.4%, respectively. Furthermore, XY1 and ZH1 significantly promoted late apoptosis effects at a concentration of 30 µg/mL. Taken together, our results suggest that XY and ZH can induce MCF-7 cells apoptosis.

### 3.7. Intracellular ROS Generation and Mitochondrial Membrane Potential (MMP) Were Decreased

**Figure 6:**
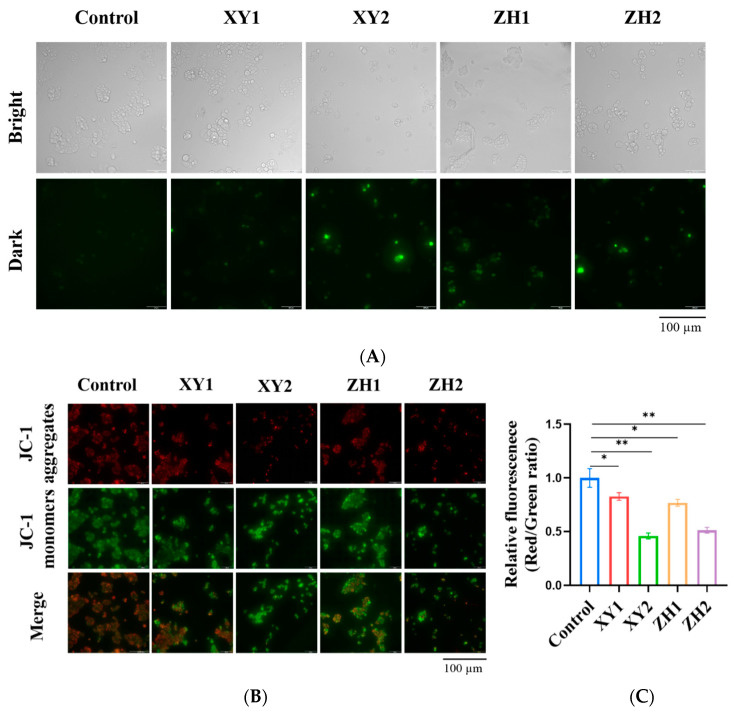
Carotenoid extracts from XY and ZH were found to induce the generation of ROS and alterations in MMP. (**A**) The images of the MCF-7 cells were captured using intracellular ROS staining using the fluorescence probe DCFH-DA. The cells were treated with XY and ZH for 48 h. (**B**) The images showing the fluorescence of MMP were captured following incubation with JC-1. The alteration in fluorescence emission, transitioning from red (JC-1 aggregates) to green (JC-1 monomers), indicates changes in Δψm. (**C**) Representative mitochondrial damage by measuring the ratio of green to red fluorescence. Mean ± SD. * *p* < 0.05; ** *p* < 0.01. XY1 = 30 µg/mL, XY2 = 60 µg/mL, ZH1 = 30 µg/mL, ZH2 = 60 µg/mL.

Excessive ROS can cause damage to biological macromolecules and trigger early apoptosis in cell lines [33]. Therefore, we determined the effect of XY and ZH on cellular ROS levels in the MCF-7 cells using a fluorescence microscope with the redox-sensitive fluorescent probe DCFH-DA. Both ZH and XY promoted the production and accumulation of ROS. After treatment with XY2 and ZH2 for 48 h, strong green fluorescence was observed in the MCF-7 cells, indicating a significant increase in ROS levels (Figure 6A). The results show that XY and ZH increased the ROS levels in breast cancer cells and severely affected cellular homeostasis.

A change or decrease in MMP is considered a significant event in apoptosis. To investigate whether XY and ZH could induce a decrease in MMPs, the MCF-7 cells were treated with XY and ZH at concentrations of 30 µg/mL and 60 µg/mL. The fluorescent images show that the XY and ZH treatments exhibited an increase in green fluorescence intensity and a decrease in red fluorescence intensity in a concentration-dependent manner (Figure 6B). Through ZH2 and XY2 treatment, the green fluorescence intensity of the JC-1 monomer was significantly increased, and the red fluorescence intensity of JC-1 aggregates was reduced. Through merging the red and green fluorescence, it became evident that the treatment group exhibited stronger green fluorescence, suggesting a lower mitochondrial membrane potential. According to our data, compared with the control group, the MMPs of XY1, XY2, ZH1, and ZH2 decreased to 0.77, 0.51, 0.83, and 0.46, respectively. Our results suggest that ZH and XY can significantly reduce MMP and affect the mitochondrial function of MCF-7 cells.

### 3.8. Effects of Carotenoid Extracts on MDA and GSH Levels in MCF-7 Cells

**Figure 7:**
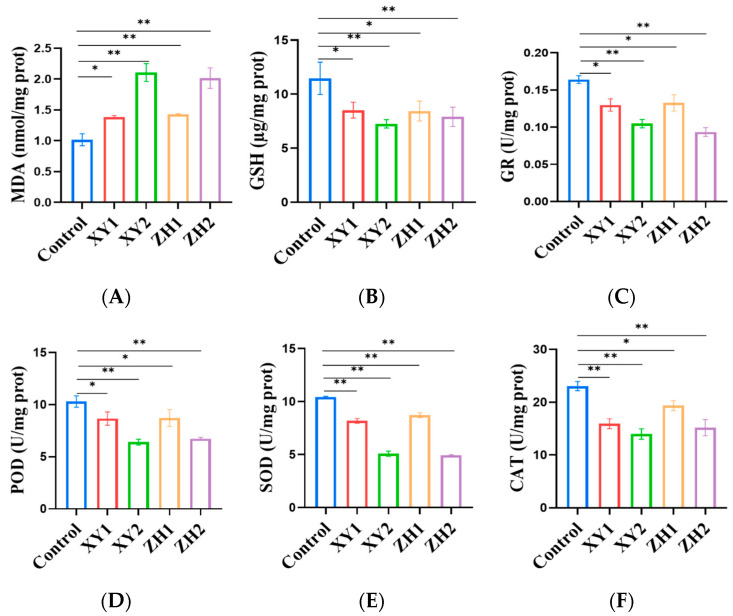
Effects of ZH and XY carotenoid extracts on MDA (**A**); GSH levels (**B**); and GR (**C**), POD (**D**), SOD (**E**), and CAT (**F**) activities in MCF-7 cells. Mean ± SD. * *p* < 0.05; ** *p* < 0.01. XY1 = 30 µg/mL, XY2 = 60 µg/mL, ZH1 = 30 µg/mL, ZH2 = 60 µg/mL.

The generation of cellular ROS leads to lipid damage, while MDA, a secondary end product of lipid peroxidation, is a marker of cellular oxidative damage [34]. Figure 7A demonstrates that the concentration of ZH1 and ZH2 in MCF-7 cells significantly increased the level of MDA by 36.27% (*p* < 0.01) and 106.86% (*p* < 0.05) at 30 and 60 µg/mL, respectively, when compared to the control group. Similarly, the MDA levels in the MCF-7 cells treated with XY1 and XY2 was higher than that of the control, indicating that XY had a significant impact on the increase in MDA levels. We can see that an increasing treatment concentration of both XY and ZH significantly increased the MDA levels, resulting in membrane lipid peroxidation.

Reduced GSH levels in tumor cells can cause mitochondrial dysfunction, leading to ROS production and, subsequently, induced cell death by apoptosis. Compared with the control, our results indicate that MCF-7 cells treated with concentrations of XY1 and XY2 for 48 h led to the statistically significant depletion of GSH levels in a dose-dependent manner. XY1 and XY2 decreased by 25.72% and 36.70%, respectively (Figure 7B). With the increase in the ZH treatment concentration in the MCF-7 cells, the GSH levels decreased significantly, and ZH1 and ZH2 decreased by 26.42% and 31.12%, respectively. XY and ZH could reduce GSH levels, but as the main compounds that maintain cell homeostasis, they may not play a significant role in scavenging O^2−^, H_2_O_2_, and lipid hydroperoxide (LOOH), indicating significantly increased oxidative stress.

### 3.9. Antioxidant Enzyme Activity of MCF-7 Cells

SOD, GR, and POD are essential components of the antioxidant enzyme system, playing a crucial role in preventing oxidative damage [35]. As shown in Figure 7C, a certain concentration of XY and ZH could significantly reduce the GR activity in MCF-7 cells. In comparison to the control group, the GR activity of XY in the MCF-7 cells decreased by 20.85% and 36.17% at 30 and 60 µg/mL, respectively. Similarly, ZH exhibited a reduction in GR activity by 13.13% and 43.00% at concentrations of 30 and 60 µg/mL, respectively. It can be seen that, in the MCF-7 cells, the higher the concentration of ZH and XY, the greater the inhibition of GR activity.

Figure 7D shows the enzymatic activity of POD at the different concentrations of ZH and XY. XY significantly decreased the POD activity in the MCF-7 cells, which decreased by 15.81% and 37.72% in XY1 and XY2, respectively. ZH1 and ZH2 also reduced the POD activity, which was significantly decreased by 15.32% and 34.45%. To summarize, both XY and ZH inhibited POD activity in the MCF-7 cells after the 48 h treatment.

As shown in Figure 7E, different concentrations of XY and ZH could significantly inhibit the SOD activity in the MCF-7 cells. Compared with the control, the SOD activity of XY was reduced by 12.98% and 45.87% at 30 and 60 µg/mL, and the SOD activity of ZH was decreased by 7.2% and 47.65% at 30 and 60 µg/mL, respectively. The SOD activity was greatly affected by XY and ZH, and the activity was significantly inhibited.

From Figure 7F, it can be observed that both XY and ZH significantly decreased the activity of CAT when compared to the control. This suggests that there is a dose-dependent relationship between XY and ZH in relation to the activity of CAT. The CAT activity of XY in the MCF-7 cells was reduced by 30.92% and 39.39% at 30 and 60 µg/mL, respectively, and the CAT activity of ZH was reduced by 16.06% and 34.15% at 30 and 60 µg/mL, respectively. After XY and ZH treatment for 48 h, the CAT enzyme activity decreased significantly.

### 3.10. Effect of Carotenoid Extracts on the Expression of Apoptosis-Related Genes in MCF-7 Cells

**Figure 8:**
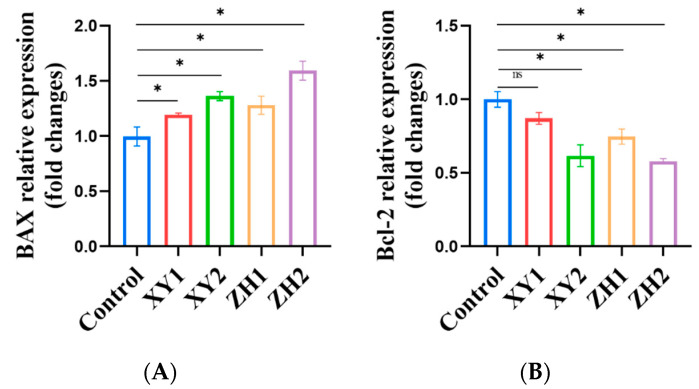

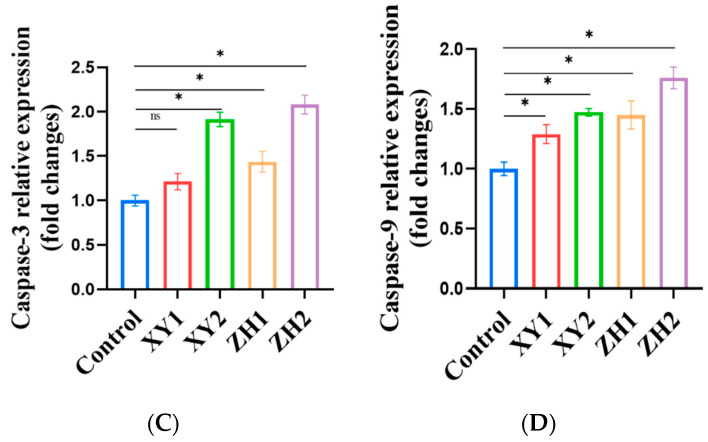
Effects of ZH and XY carotenoid extracts on cancer-related gene expression in MCF-7 cells. (**A**) Bax; (**B**) Bcl-2; (**C**) Caspase-3; (**D**) Caspase-9. Mean ± SD. * *p* < 0.05. XY1 = 30 µg/mL, XY2 = 60 µg/mL, ZH1 = 30 µg/mL, ZH2 = 60 µg/mL. ns means no significant difference in statistics.

To further elucidate the mechanism by which carotenoid extracts induce apoptosis, we investigated the pro-apoptotic effects of XY and ZH in the MCF-7 cancer cells. We analyzed the gene expression levels of apoptosis marker genes Bcl-2 and Bax using qRT-PCR. As can be seen in Figure 8, the gene expression of Bax was significantly increased in a dose-dependent manner following the XY and ZH treatments. Conversely, the gene expression of Bcl-2 was significantly decreased. In the MCF-7 cells exposed to 60 µg/mL of XY2, the expression of Bax, associated with mitochondrial activity, increased by 1.37 ± 0.09-fold, while the expression of Bcl-2 decreased by 0.62 ± 0.03-fold. In the MCF-7 cells treated with 60 µg/mL of ZH2, the expression of Bax showed an increase of 1.58 ± 0.10-fold, while Bcl-2 exhibited a decrease of 0.58 ± 0.05-fold. In comparison to the control group, we noticed a remarkable increase in the gene expression of caspase-3 in a dose-dependent manner following the XY and ZH treatments. Caspase-3 expression increased 1.22 ± 0.08-fold and 1.91 ± 0.10-fold after treatment with XY1 and XY2, respectively, and it increased 1.44 ± 0.09-fold and 2.08 ± 0.11-fold after treatment with ZH1 and ZH2, respectively. Similarly, the gene expression of caspase-9 increased in a concentration-dependent manner with the increase in treatment concentration. Caspase-9 expression increased 1.47 ± 0.08-fold and 1.76 ± 0.09-fold after treatment with XY2 and ZH2, respectively, compared to the untreated cells.

## 4. Discussion

As is known, breast cancer is a prevalent and harmful malignant disease affecting women worldwide, regardless of the country’s development status. Consequently, there is a growing interest in exploring natural products as potential cancer treatments. Carotenoids, known for their strong antioxidant properties, have demonstrated promising effects in cancer prevention. Citrus fruits are a diverse source of carotenoids and have the highest carotenoid content of all fruits [36]. Consistent with the pulp color phenotype of five citrus fruits, the color of the carotenoid extracts was also found to be significantly different. This variation in color among the citrus cultivars can be attributed to differences in carotenoid content and composition [37]. Consistent with previous research, this study found that the pulp extracts contained a large amount of β-cryptoxanthin and lutein in all species, except for XY [28,38]. In the case of XY, the carotenoid compositions mainly contained lycopene and beta-carotene. A previous study demonstrated the significant genetic variations in fruit color and carotenoid pigments among different citrus groups and even within varieties [39]. Additionally, it has been reported that environmental conditions can influence the composition and content of carotenoids in citrus fruits [40]. Therefore, carotenoids are not only affected by genetic factors but also by environmental factors. The antioxidant capacity of citrus carotenoid extracts was assessed via common methods used in foods, which included the ABTS, DPPH, and FRAP radical scavenging assays. ZHQC exhibited the highest antioxidant activity, with a TEAC of 2.44 µmol TE/g FW, 6.85 µmol TE/g FW, and 2.77 µmol TE/g FW (obtained using the DPPH, ABTS, and FRAP methods, respectively). However, NFMJ showed the lowest antioxidant capacity following the application of the DPPH, ABTS, and FRAP radical scavenging activity methods. The ABTS value for the antioxidant capacity was found to be higher than the DPPH and FRAP values. This is due to ABTS having a wide range of PH applications, a short detection reaction time, and good solubility [10,41]. Carotenoids are easily soluble in hydrophobic solvents, so the ABTS value is higher than that of DPPH and FRAP in terms of the total antioxidant capacity. After evaluation using the comprehensive antioxidant performance index (APC), the citrus fruits were ranked as follows: ZHQC > DFHJ > ZMPG > XY > NFMJ. Another study ranked the antioxidant capacity of carotenoid monomers as follows: lycopene > α-carotene > β-carotene > β-cryptoxanthin > zeaxanthin [10]. Thus, the antioxidant activity of citrus fruits is affected by the combined effects of carotenoid content and composition, which may provide consumers with good health benefits.

There is increasing evidence to suggest that carotenoids exhibit promising pharmacological effects in cancer therapies, including anti-inflammatory, anti-proliferation, and pro-apoptosis effects, as well as activities that disrupt the cell cycle [22,42]. In this study, our objective was to investigate the impact of carotenoid extracts in regard to anti-proliferation, their oxidative status (including the generation of ROS), and the signaling pathway of apoptosis in MCF-7 cells. In comparison to the other carotenoid extracts (ZMPG, NFMJ, and DFHJ), the viability of the MCF-7 cells was significantly inhibited by the ZH and XY treatments. Studies have shown that the stronger inhibitory effect of carotenoid mixtures on MCF-7 cell proliferation may be due to their synergistic effect [21]. Another study showed that a combination of carotenoids effectively inhibited the activity of PC-3 prostate cancer cells and MCF-7 breast cancer cells [43]. Moreover, cell viability showed a concentration-dependent decrease with increasing treatment concentrations, indicating that higher concentrations have a greater cytotoxic effect on cells. The infiltration and metastasis of breast cancer cells are some of the most important causes for the deterioration of cancer patients [44]. It is a complex process that includes cancer cell adhesion, invasion, and migration. Several studies have shown that carotenoid extracts can inhibit the growth and migration of cancer cells [45,46,47]. Consistent with the findings of previous studies, our study showed that carotenoid extracts can significantly inhibit the migratory ability of MCF-7 cells. Through an Edu anti-proliferation assay and colony formation assay, we found that ZHQC and XY significantly inhibited cell growth by decreasing the number of viable MCF-7 cells. Similarly, it has been shown that sweet potato carotenoid extract can effectively inhibit the growth of human breast cancer MCF-7 cells and tumors in mice [20]. Poorigali Raghavendra-Rao Sowmya et al. also showed that an equimolar concentration of a carotenoid mixture can effectively inhibit cell activity [22]. These findings suggest that carotenoid extracts have anti-proliferative and anti-migratory effects on breast cancer cells.

Intracellular ROS is a key mediator of cancer proliferation and apoptosis, which plays a role in regulating cellular homeostasis [48]. Further studies have shown that the increased ROS and altered redox status observed in tumor cells can induce apoptosis [49]. This biochemical basis has recently attracted attention as a potential therapeutic method, especially for cancer cells that are susceptible to oxidative stress caused by exogenous ROS-generating agents that generate ROS and disrupt their own antioxidant system [50]. In our study, the carotenoid extracts induced the excessive accumulation of intracellular ROS in the MCF-7 cells, which affected the redox homeostasis and even induced apoptosis through oxidative stress in the MCF-7 cells. Lycopene oxidative products have been found to increase reactive oxygen species (ROS) levels and induce apoptosis in human prostate (PC-3), breast (MCF-7), and cervical cancer cells [51]. Moreover, previous studies have shown that carotenoids can increase ROS production and affect cell viability [52,53]. The excessive production of ROS can result in the breakdown of lipid peroxidation into MDA, a hallmark of oxidative stress [35]. After treatment with carotenoid extracts, MDA levels were observed to increase compared to the control group. Similarly, ROS in cells is degraded by the antioxidant GSH, and a reduction in its level is a key part in breast cancer cell apoptosis [54,55]. We observed that MCF-7 cells exhibited decreased GSH levels, indicating a significant increase in oxidative stress. The crucial role of the antioxidant enzyme system lies in maintaining redox homeostasis and minimizing the impact of oxidative stress (mainly caused by free radicals) [56]. SOD serves as the initial line of defense against ROS, and we observed that SOD activity decreased after carotenoid extract treatment. GR can reduce GSSG to regenerate GSH, which is crucial in the prevention of oxidation. Furthermore, SOD has the capability to transform a superoxide anion into hydrogen peroxide, whereas CAT facilitates the conversion of hydrogen peroxide into water and oxygen, thus diminishing the intracellular ROS concentration [57]. However, the carotenoid extracts, especially XY2 and ZH2, reduced the SOD, CAT, POD, and GR activities in the MCF-7 cells, affecting cell homeostasis and inducing oxidative damage. According to previous studies, carotenoid-oxidized products exhibit a decline in the viability of cancer cells, and this is accompanied by elevated levels of MDA and ROS, as well as reduced levels of GSH [53]. In conclusion, our results demonstrate that carotenoid extracts exert anticancer effects by enhancing the levels of pro-oxidant factors (ROS and MDA) and reducing the activity of antioxidant enzymes (SOD, GSH, CAT, SOD, and POD) in cells.

For normal cell growth, the equilibrium is maintained between cell proliferation and apoptosis. Uncontrolled cell growth is the basis of tumorigenesis, which mainly manifests as destroying cell proliferation and interfering with cell apoptosis. The pro-oxidation effects of carotenoids may be beneficial in the fight against cancer. In cancer cells, the pro-oxidative activity of carotenoids produces oxidative damage, inhibiting cancer cell proliferation and limiting cancer progression and metastasis [16]. In our study, the cell cycle arrest in the G0/G1 phase, in conjunction with the reduction in the proliferation of carotenoid extract-treated cells, indicates that carotenoid extracts have cytostatic effects on the human breast cell line MCF-7. This is consistent with a study by Upadhyaya K R et al., who showed that carotenoids are involved in cell cycle arrest, leading to increased accumulation in the G0/G1 phase [58]. These findings are consistent with previous reports on other carotenoids, such as lycopene and vitamin D3, which synergistically disrupt the G0/G1 phase of the cell cycle [59]. As the cell cycle progresses, arrest in the G1 phase can result in apoptosis or the activation of repair mechanisms. Several studies have shown that carotenoids inhibit cancer cell proliferation by inhibiting the expression of cyclin D1, thereby preventing the progression of the G0/G1 cell cycle [23,60,61]. Shrimp carotenoid extract can also effectively induce cell cycle arrest and apoptosis after treatment with a carotenoid mixture (keto-carotenoid astaxanthin plus β-carotene and lutein), indicating the synergistic effect of carotenoids [22,62]. In summary, carotenoid extracts derived from XY and ZH can arrest the cell cycle and induce apoptosis to achieve anti-breast cancer effects.

Increased oxidative stress causes mitochondrial damage, which can trigger apoptosis [63]. One of the key events in early apoptosis is an alteration or decrease in cellular mitochondrial membrane potential [64]. With an increase in the concentration of the carotenoid extract treatment, we observed a decrease in the mitochondrial membrane potential, indicating that apoptosis occurred in these cells. Apoptosis is the process of programmed cell death, and it involves intrinsic (mitochondrial) and extrinsic (ligands and death receptors) molecular pathways. Apoptosis induced by chemotherapy is largely regulated by mitochondria and Bcl-2 family proteins, which exert anti-apoptotic and pro-apoptotic effects by acting on the mitochondria [65,66]. This study showed that carotenoid extracts increase Bax gene expression and decrease Bcl-2 expression, resulting in apoptosis. Bax promotes mitochondrial membrane permeability, leading to a subsequent caspase apoptotic cascade, whereas Bcl-2 blocks this process [67]. The caspase family is closely related to the apoptotic process and includes caspase-3, 7, and 9, of which caspase-3 is considered to be the “executor” in the apoptotic process [68]. The results from our qRT-PCR analysis showed that the carotenoid extracts significantly increased the gene expression of caspase-3 and 9. As we know, these genes are mainly involved in the intrinsic pathway of apoptosis. Therefore, these findings suggest that carotenoid extracts can induce breast cancer cell apoptosis via the mitochondrial apoptosis pathway.

## 5. Conclusions

In summary, our study showed that citrus pulp extracts are rich in carotenoids. The XY fruit pulp extract contains the highest carotenoid content, while the ZH fruit pulp extract exhibits the strongest antioxidant capacity. Carotenoid extracts from ZH and XY demonstrated inhibitory effects on MCF-7 breast cancer cells through the suppression of cell proliferation, cell cycle arrest, and the induction of apoptosis. The carotenoid extracts of ZH and XY induce oxidative stress and regulate apoptosis in MCF-7 cancer cells through their pro-oxidative potential. Carotenoid extracts have the ability to trigger the mitochondrial-dependent intrinsic apoptosis pathway, thereby assisting in preventing potential drug resistance in cancer cells. Carotenoid-rich extracts have been found to exhibit pro-oxidant activity. Based on the long history of carotenoids being used as food additives and their anti-proliferative effects against human MCF-7 breast cancer cells, carotenoid-rich extracts are promising nutraceuticals and dietary supplements that could be used in cancer therapies.

## Figures and Tables

**Table 1:** The details of the citrus materials.

No.	Citrus Resources	Latin Name	Locations	Abbreviation
1	ZaoMiPengGan	*Citrus reticulata* Blanco cv. Ponkan	Xiangxi, Hunan	ZMPG
2	DongFangHong	*Citrus reticulata* Blanco cv. DongFangHong	Nanfeng, Jiangxi	DFHJ
3	NanFengMiJu	*Citrus reticulata* Blanco cv. Kinokuni	Nanfeng, Jiangxi	NFMJ
4	XiYou	*Citrus paradisi* Macf.	South Africa	XY
5	ZaoHongQiCheng	*Citrus sinensis* Osbeck cv. ‘ZaoHong’	Zigui, Hubei	ZHQC

**Table 2:** The basic information of detected 9 carotenoids from citrus fruit.

Name	Retention Time (min)	Peaks	Chemical Structure	Molecular Formula
lutein	20.34	445.7,472.4	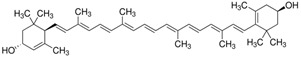	C_40_H_56_O_2_
Zeaxanthin	21.60	451.8,475.9	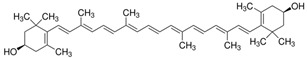	C_40_H_56_O_2_
β-cryptoxanthin	27.73	451.8,479.7	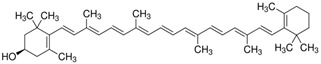	C_40_H_56_O
α-carotene	32.87	446.9,474.8	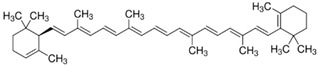	C_40_H_56_
β-carotene	35.11	453.0,479.7	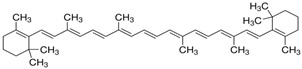	C_40_H_56_
Lycopene	55.16	473.6,505.2	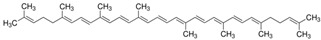	C_40_H_56_
violaxanthin	16.15	438.6,467.5	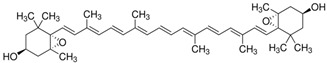	C_40_H_56_O_4_
9-cis-violaxanthin	18.36	436,463.9	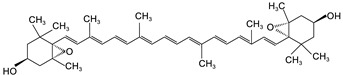	C_40_H_56_O_4_
Luteoxanthin	17.31	422.7,448.1	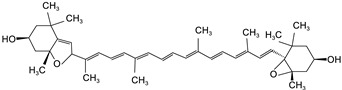	C_40_H_56_O_4_

**Table 3:** Antioxidant capacities of the citrus carotenoid extracts.

Citrus Resources	DPPH (μmol/g TE FW)	ABTS (μmol/g TE FW)	FRAP (μmol/g TE FW)	APC	Rank
DFHJ	2.02 ± 0.13 ^b^	5.31 ± 0.16 ^b^	2.35 ± 0.01 ^b^	81.64	2
NFMJ	1.07 ± 0.04 ^d^	2.91 ± 0.33 ^d^	1.49 ± 0.09 ^c^	46.70	5
ZHQC	2.44 ± 0.1 ^a^	6.85 ± 0.02 ^a^	2.77 ± 0.09 ^a^	100.00	1
ZMPG	1.63 ± 0.13 ^c^	3.75 ± 0.16 ^c^	2.19 ± 0.14 ^b^	66.74	3
XY	1.85 ± 0.22 ^bc^	3.11 ± 0.12 ^d^	2.17 ± 0.14 ^b^	66.39	4

Values are expressed as mean ± standard deviation (*n* = 3). Statistical evaluation was performed using the Tukey comparison test. Bars with different letters (a–d) are significant (*p* < 0.05).

**Table 4:** IC50 values (µg/mL) of carotenoid extracts in MCF-7 cell lines.

Carotenoid Extracts Semi-Inhibitory Concentration					
	DFHJ	NFMJ	ZHQC	ZMPG	XY
IC50 (µg/mL)	91.70	86.17	58.53	68.78	61.34

## Data Availability

The datasets generated for this study are available on request to the corresponding author.

## Supplementary Materials

The following supporting information can be downloaded at: https://www.mdpi.com/article/10.3390/foods12183469/s1, Table S1: Information of Gene Primer Sequences.
